# Biodegradable and Biocompatible Silatrane Polymers

**DOI:** 10.3390/molecules26071893

**Published:** 2021-03-26

**Authors:** Vladislav V. Istratov, Valerii A. Vasnev, Galy D. Markova

**Affiliations:** Nesmeyanov Institute of Organoelement Compounds, Russian Academy of Sciences, 119991 Moscow, Russia; vasnev@ineos.ac.ru (V.A.V.); m-galy@mail.ru (G.D.M.)

**Keywords:** biodegradable polymers, branched block copolymers, silatrane, amphiphilicity, surface activity, biological activity

## Abstract

In this study, new biodegradable and biocompatible amphiphilic polymers were obtained by modifying the peripheral hydroxyl groups of branched polyethers and polyesters with organosilicon substituents. The structures of the synthesized polymers were confirmed by NMR and GPC. Organosilicon moieties of the polymers were formed by silatranes and trimethylsilyl blocks and displayed hydrophilic and hydrophobic properties, respectively. The effect of the ratio of hydrophilic to hydrophobic organosilicon structures on the surface activity and biological activity of macromolecules was studied, together with the effect on these activities of the macromolecules’ molecular weight and chemical structure. In particular, the critical micelle concentrations were determined, the effect of the structure of the polymers on their wetting with aqueous solutions on glass and parafilm was described, and the aggregation stability of emulsions was studied. Finally, the effect of the polymer structures on their antifungal activity and seed germination stimulation was examined.

## 1. Introduction

Biodegradable polymers are successfully used in medicine due to their ability to easily adjust properties, such as molecular weight, local microstructure, degradation rate, mechanical strength and stiffness. These polymers can be used singly or as composites. Polymer devices made from them usually represent a good compromise between biocompatibility and mechanical requirements. Biodegradable polymers are used in vivo as medical devices, such as implants and prostheses, sutures, drug-eluting stents, and drug delivery devices [1,2,3,4]. One of the very important biomedical applications of such polymers is the preparation of colloidal drug delivery systems. With advantages, such as increased solubility, bioavailability, and the ability to target drug delivery and release to specific locations, this drug transport method is at the forefront of current research [3]. Several types of colloidal systems have been developed, investigated, and applied in clinical practice, including micelles, liposomes, nanocarriers, liquid crystalline phases, aerosols, microemulsions, and procolloidal systems. All of them are based on the use of surfactants, often polymeric ones. [5,6] Nonionic surfactants are mainly used in medicine, although anionic, cationic and zwitterionic analogs are also applied [7]. Due to the uncharged nature of nonionic surfactants, they are less sensitive to the acidity of the environment but instead rather sensitive to temperature, which can be used as a trigger for drug delivery. The critical micelle concentration for such surfactants is usually much lower than for the corresponding charged ones, and partly because of this, such substances are usually less irritating and better tolerated than anionic and cationic compounds [6]. New polymeric surfactants with low toxicity and high biodegradability, especially from renewable sources, have received a powerful boost over the past two decades. In particular, nonionic polymers containing hydrophobic blocks formed by polymers of amino and hydroxy acids are of great interest since their decomposition leads to the formation of non-toxic, water-soluble oligomers and natural metabolic products [8]. Currently, the most fully studied biodegradable polymeric surfactants have a linear structure, although branched surfactants can have advantages in a number of properties and areas of application [6,9,10,11,12]. Unfortunately, there is currently insufficient experimental data for the targeted synthesis of branched polymeric surfactants with desired properties. Recently, a number of scientists [13,14,15,16,17] have drawn attention to the synthesis and study of the so-called surfadrugs, that is, amphiphilic substances with covalently bound drug molecules. Fragments of the drug in these substances serve as hydrophilic or hydrophobic blocks while simultaneously imparting biological activity to the entire molecule. In this work, silatrane, a pentacoordinated silicon chelate, was used to prepare polymeric surfadrug. A detailed study of silatranes was begun in the middle of the last century by academician M.G. Voronkov [18]. The unique antiviral, anti-inflammatory, antitumor, antibacterial and antifungal effects of silatranes, as well as the demonstrated stimulating effect on the productivity of animals and plants [18,19,20,21,22], ensured the use of these substances in medicine, cosmetology and agriculture. To date, various silatranes with various substituents have been synthesized and studied both in the silatrane cycle itself and at the silicon atom. One of these compounds is 1-(3-aminopropyl)silatrane, which has a wide spectrum of action–from antimicrobial, antiviral, anticancer action to stimulating plant growth. Unfortunately, to date, the overwhelming majority of works devoted to silatranes are focused on the biological activity of low molecular weight compounds. There are practically no publications on the biological activity of silatrane-containing polymers. The few articles devoted to polymer silatranes [23,24,25,26,27] concern only the synthesis and study of the physical properties of these macromolecules. As far as we know, there is practically no literature on the surface of bioactive properties of silatrane-containing polymers. In this regard, the synthesis of amphiphilic macromolecules containing 1-(3-aminopropyl)silatrane is of great theoretical and practical interest in terms of obtaining new branched surface and biologically active polymers and expanding the spectrum of available pharmacological compositions. This article is devoted to the synthesis and study of such polymers.

## 2. Results

### 2.1. Polymer Structure Elucidation

**Figure 1:**
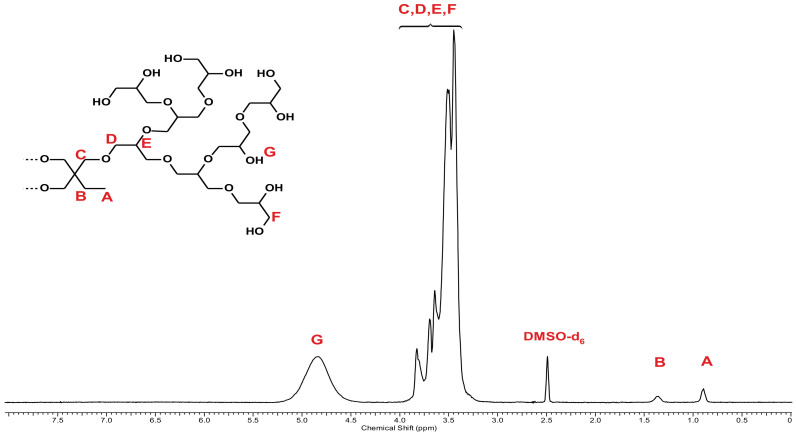
1H-NMR spectrum and schematic architecture of polyglycerol.

**Figure 2:**
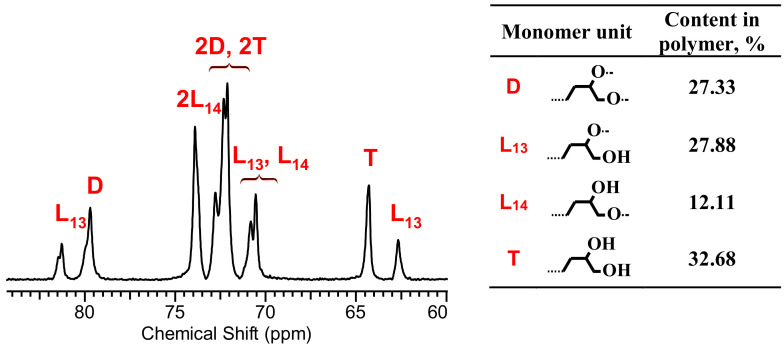
^13^C NMR inverse gated spectrum of polyglycerol and content of different units in the polymer. Carbons belonging to the dendritic, linear 1,3, linear 1,4 and terminal, units are indicated by D, L_13_, L_14_, and T, respectively.

The polyethers and polyesters used in this work have a similar configuration; that is, they are formed by the addition of monomeric molecules to a central 1,1,1-tri(hydroxymethyl)propane molecule (Figure 1):

Hence, the ^1^H-NMR spectra of these polymers are also quite simple and contain signals of elementary units and 1,1,1-tri(hydroxymethyl)propane (Figure 1; Figure 2). In particular, the spectrum of polyglycerol (Figure 1) contains signals of methyl and methylene groups of 1,1,1-tri(hydroxymethyl)propane (0.85 and 1.35 ppm, respectively), as well as a broad signal of methylene and methine groups of elementary polymer units (3.10–4.0 ppm). Comparing the integral intensities of the signals of methyl groups of 1,1,1-tri(hydroxymethyl)propane and methyl with methylene groups of the elementary polymer units, the degree of polymerization was found to be 56, and the average functionality of the macromolecule was equal to 59. Since the integrated signal intensities of 1,1,1-tri(hydroxymethyl)propane of the methyl and methylene groups are low, we additionally calculated the degree of polymerization using the ^13^C NMR inverse gated spectrum by the technique presented in [28]. Integrated intensities of signals corresponding to dendritic (D), linear(L_13_ and L_14_) and terminal (T) groups were obtained from NMR spectra, and their relative content in the polymer was determined (Figure 2).

Then, according to the equation given in [28]:

DP_n_ = f_c_ (T + L_13_ + L_14_ + D)/(T − D) (1)

where DP_n_ represents the number–average degree of polymerization, f_c_ is the functionality of the core molecule, and T, L_13_,L_14_,D designates the corresponding monomer units.

The DP_n_ of the polymer was determined and found to be 56. Thus, calculations using both ^1^H NMR and ^13^C NMR produce similar results.

**Figure 3:**
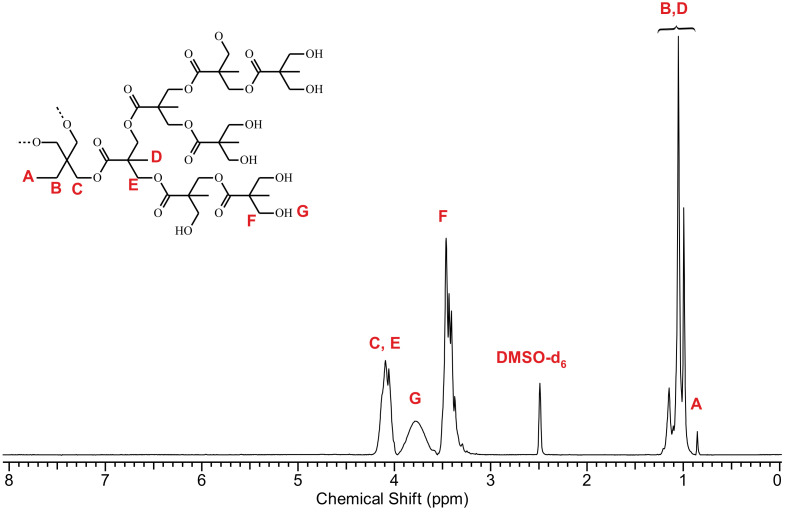
1H-NMR spectrum and schematic architecture of Boltorn H40.

The ^1^H-NMR spectrum of Boltorn H40 polyester (Figure 3) includes signals of methyl groups of 1,1,1-tri(hydroxymethyl)propane (0.85 ppm), a set of signals of methylene groups of 1,1,1-tri(hydroxymethyl)propane and methylene and methyl groups of the elementary polymer units (0.95–1.25 ppm), and several sets of signals corresponding to the terminal methylene groups (3.15–3.55 ppm) and those of the polymer chain (3.95–4.25 ppm) of the elementary polymer units.

Additionally, the spectrum includes a broad signal of free hydroxyl groups at 3.55–3.9 ppm. Comparing the integral intensities of signals at 0.85 ppm and 0.95–1.25 ppm, the degree of polymerization was found to be 61, and the average functionality of the macromolecule was 64, which is consistent with the manufacturer’s declared 64 end-OH groups. Thus, it can be concluded that both polymers have similar degrees of polymerization.

**Figure 4:**
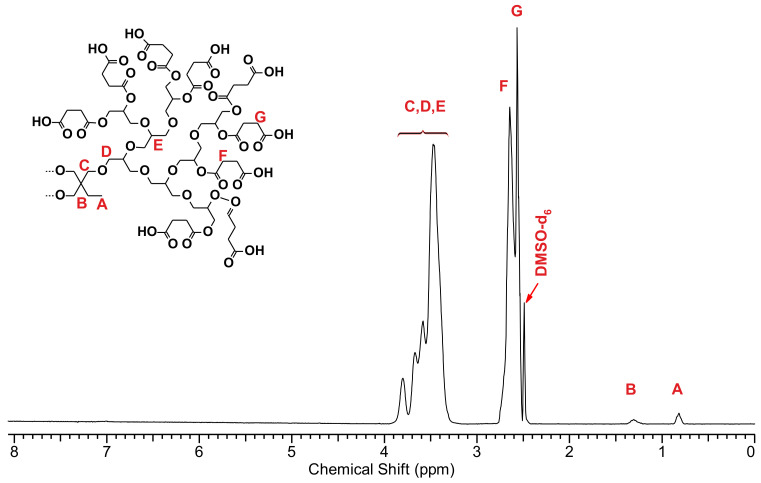
^1^H-NMR spectrum and schematic architecture of carboxy-functionalized polyglycerol.

Both polyglycerol and Boltorn H40 were further carboxy-functionalized, using the excess of succinic anhydride, the reaction progression being monitored by ^1^H-NMR: For polyglycerol, the integral intensities of the signals corresponding to methylene and methine groups of the elementary polymer units and methylene groups of succinic acid were compared (Figure 4).

**Figure 5:**
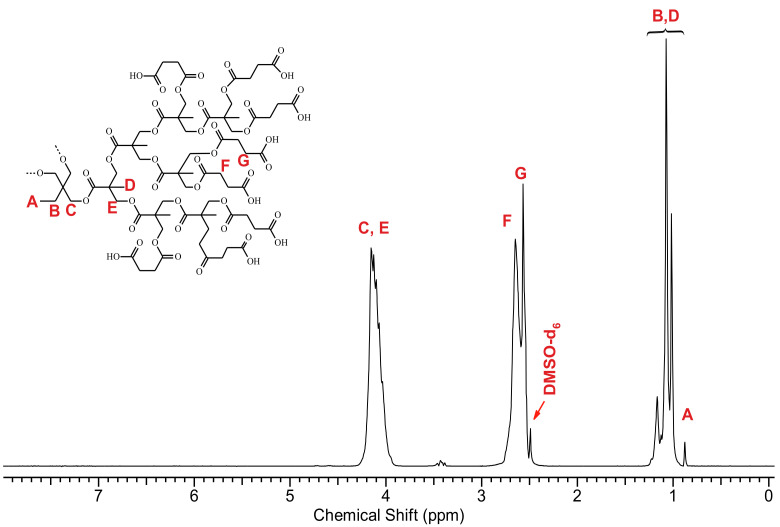
1H-NMR spectrum and schematic architecture of carboxy-functionalized Boltorn H40.

For Boltorn H40, the integral intensities of the signals corresponding to methylene groups of the polymer and succinic acid were compared (Figure 5).

For both polyglycerol and Boltorn H40 polymers, the degree of the terminal hydroxyl group substitution was found to be 99.4–99.7%. This fact, together with the disappearance of the signals of terminal OH groups, allowed us to conclude that the hydroxyl groups of the polymers were practically completely substituted.

**Figure 6:**
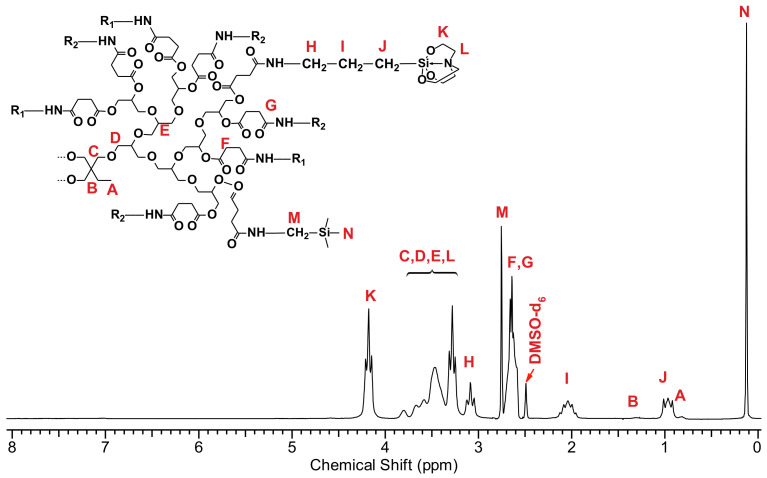
1H-NMR spectrum and schematic architecture of silatrane-containing polyglycerol.

**Figure 7:**
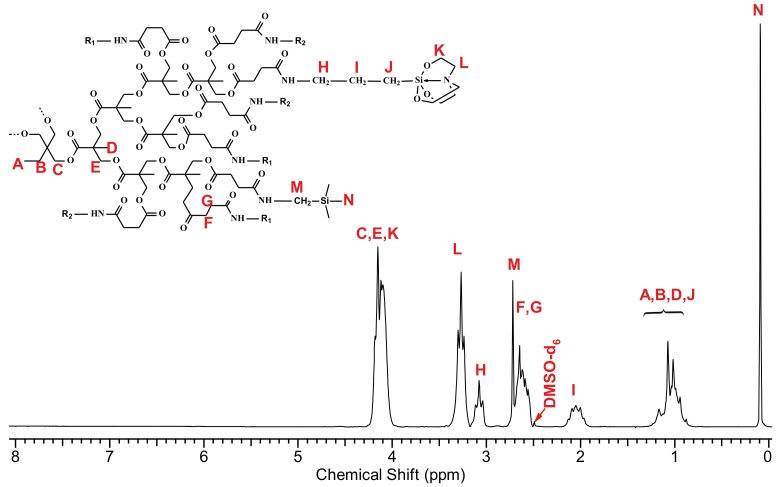
1H-NMR spectrum and schematic architecture of silatrane-containing Boltorn H40.

At the final stage of the synthesis, these compounds were sequentially reacted with 1-(3-aminopropyl)silatrane and (aminomethyl)trimethylsilane to form polymers 3 and 4. (Figure 6; Figure 7, respectively):

Substitution of free carboxyl groups was monitored by ^1^H-NMR, and for both polyglycerol and Boltorn H40, the integral intensities of the signals were compared, corresponding to methylene groups of silatrane, methylene groups of succinic acid and methyl and methylene groups of (aminomethyl)trimethylsilane.

The polymers, which were obtained in the form of a white, waxy substance soluble in dimethylsulfoxide (DMSO) and tetrahydrofurane (THF), differed in their polymer core composition and silatrane group content. An increase of the silatrane content in the polymers resulted in an increased rate of solubility of the latter in methanol and water, thus changing from insoluble to completely soluble. All the polymers were characterized by NMR spectroscopy and GPC. The composition of the polymers was monitored by ^1^H-NMR spectroscopy (Table 1). It is evident that the calculated and experimentally determined amounts of silatrane-substituted carboxyl groups are similar, indicating that the resulting structures correspond to the calculated ones. Thus, we synthesized polymers with a branched core formed by polyethers (polymers **3a**–**d**) or polyesters (polymers **4a**–**d**); polymers **3a**–**d** and **4a**–**d** differed by the structure of the branched block, with the ratios of R_1_ to R_2_ substituents (see experimental part Schemes 3 and 4) in these polymers’ series changing analogously.

**Figure 8:**
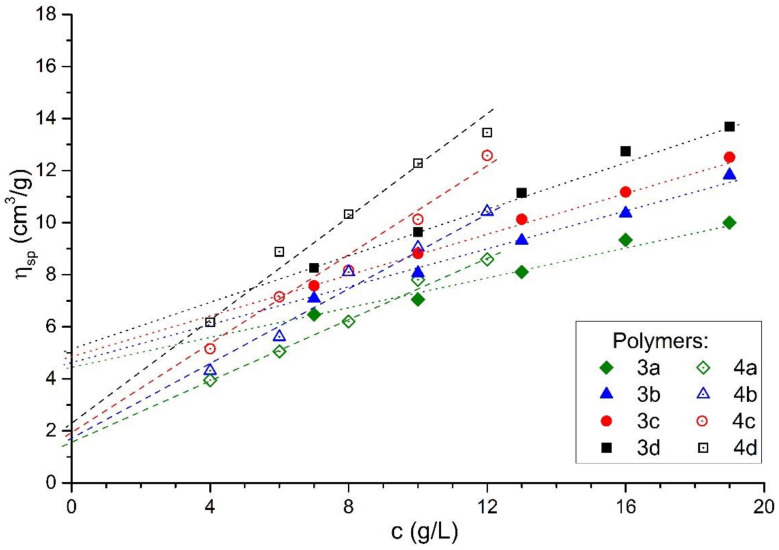
Specific viscosity of silatrane-containing polymers as a function of concentration in tetrahydrofurane at 25 °C.

To obtain additional information on the studied polymers’ behavior in solutions, their intrinsic viscosities were determined in THF. Figure 8 shows the relationship between the specific viscosity of polymers **3a–d** and **4a–d** at various concentrations.

The linearity of the plots shows that none of the polymers formed aggregates in solution. The intrinsic viscosity ([η]) of the polymers was determined from a plot of specific viscosity vs. concentration; the values are shown in Table 1. Polyester polymers have the lowest [η] values (1.49–2.31 dL/g) at 25 °C compared to polyether polymers (4.40–5.25 dL/g). This is likely due to the more compact structure of Bolthorn compared to polyglycerol. The intrinsic viscosity of all polymers based on the same branched polymer is close and slightly increases with an increase in the content of silatrane fragments. The reduced viscosity values of polymers 3a–d and 4a–e are close to the viscosity of the corresponding unsubstituted polyglycerol and Bolthorn [28,29].

### 2.2. Polymer Properties

**Figure 9:**
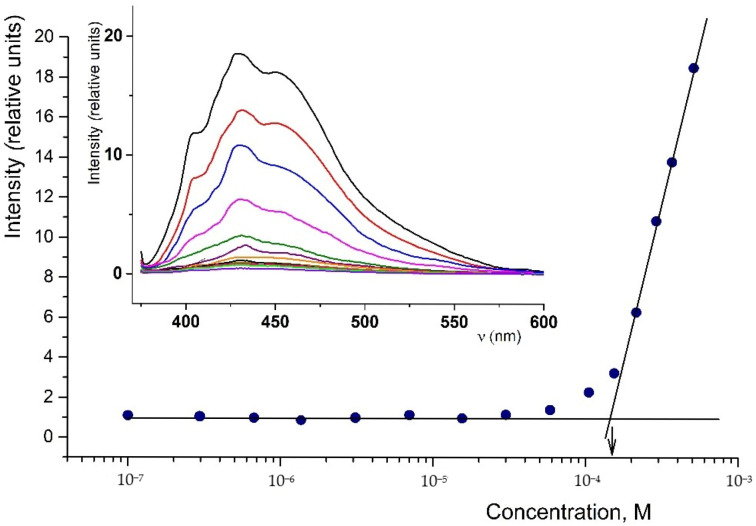
Determination of critical micelle concentration (CMC) values for polymer **3a**. Colored lines are fluorescence intensity data obtained for polymer solutions of various concentrations; on their basis, a diagram of intensity vs. concentration was drawn up. The arrow shows CMC.

Since all the polymers synthesized in this study consisted of hydrophilic and hydrophobic parts, we expected them to behave amphiphilically and, thus, to possess a surface activity. Thus, we examined micelles formation from these polymers in aqueous solutions. For this purpose, the critical micelle concentration (CMC) values of these polymer solutions were determined by fluorescence quenching (Figure 9).

The results obtained are presented in Table 2, along with the hydrophilic/lipophilic balance (HLB) values calculated using the Griffin equation. For all the studied polymers, an increase in the content of silatrane fragments in the macromolecule leads to an increase in HLB and CMC values.

Within each series of polymers (**3a**–**d, 4a**–**d**), CMC increased with an increase of HLB. However, when comparing polymers with a similar silatrane content belonging to different series, a stable difference in CMC was observed: polyether-based polymers had higher CMC values. This is probably due to the greater solubility of the polyether core of the macromolecules in water.

**Figure 10:**
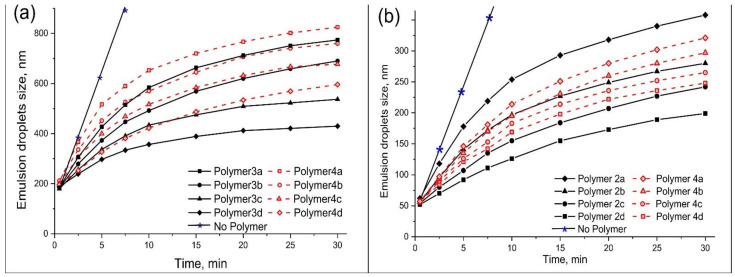
Change of the droplet sizes of emulsions stabilized by silatrane polymers. (**a**) direct emulsions, (**b**) reverse emulsions. Curve * not stabilized emulsion.

Since the formation of stable emulsions may be one of the desirable properties of polymeric surfactants, we tested the potential role of the polymers as stabilizers of direct (“oil in water”) and reversed (“water in oil”) emulsions. The droplet size of direct emulsions was shown to significantly exceed the droplet size of reverse emulsions in the presence of each of the studied polymers (Figure 10).

In the absence of the emulsion stabilizers, phase separation for both direct and reverse emulsion occurred within 10 min. At the same time, in the presence of the studied polymers, after 30 min, the size of the droplets of direct emulsions increased by only 2–4 times, while those of reverse emulsions increased by 3–8 times.

The aggregative stability of direct and reverse emulsions depended on the content of silatrane fragments, as well as on the structure of the branched core blocks. Thus, aggregative stability of direct emulsions (Figure 10a) increased with an increase of the silatrane content in a polymer. For polymers with a similar silatrane content, polyether-based polymers create more stable direct emulsions. The aggregative stability of reverse emulsions (Figure 10b) increased with a decrease of silatrane content in a polymer. Surprisingly, for polymers with a similar silatrane content, polyether-based polymers, again, formed more stable emulsions.

For both direct and reverse emulsions, polyester-based polymers form emulsions, the aggregative stability, which is less sensitive to the silatrane content in a polymer.

One of the most important characteristics of amphiphilic polymers is their ability to wet solid surfaces of varying hydrophobicity. Wettability is normally determined by measuring the contact angle between a solid surface and a surfactant solution. The contact angle is the main parameter that characterizes the drop shape on the solid surface and is also one of the properties of the phase interface that can be measured directly. It was found (Table 3) that all the silatrane polymers studied here were able to increase the wettability of both hydrophilic (glass) and hydrophobic (Parafilm) surfaces.

The solutions of all the polymers studied here displayed a partial surface wettability (contact angle θ < 90°) of both substrates (glass and parafilm). With an increase of hydrophilicity, the wettability of the glass surface increased, while the wettability of the parafilm surface decreased. Higher glass wettability was observed for polyether-based polymers, and higher parafilm wettability was observed for polyester-based polymers. For all the polymers studied, the contact angle tended to decrease with a CMC increase; however, we were unable to establish a mathematical relationship between these parameters.

**Figure 11:**
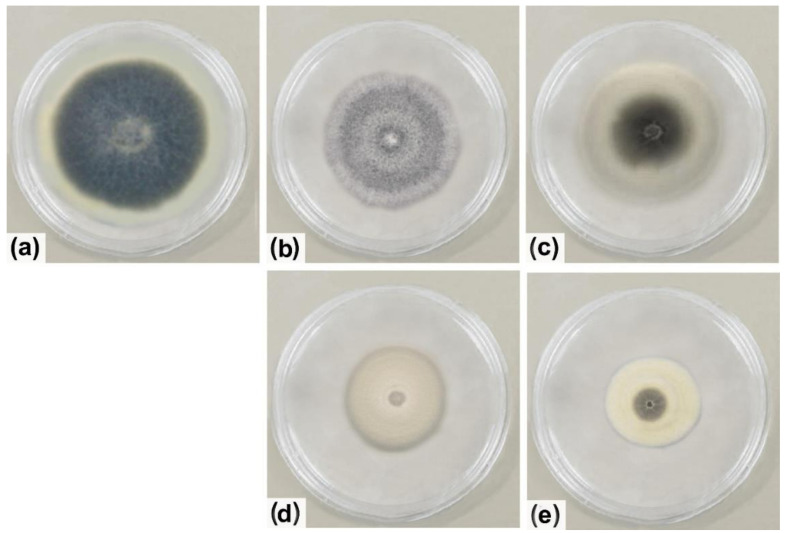
Influence of silatrane polymers on the growth of *Verticillium dahliae* colonies. (**a**) control colony; (**b**) polymer 3c; (**c**) polymer 4c; (**d**) polymer 3d; (**e**) polymer 4d. The time after inoculation is 7 days.

It is known that the biological activity of silatranes is determined primarily by the structure of their Si-substitute. In particular, silatranes with an amino group in the aliphatic substitute of a silicon atom can possess the antifungal activity and affect seed germination [21,22,30]. Hence, it was important to evaluate the biological activity of silatrane-containing polymers. First, we focused on testing the antifungal activity of the obtained silatrane-containing polymers against the phytopathogenic fungi *Verticillium dahliae*. The results are presented in Figure 11 and Table 4.

Our preliminary results showed that the polymers with maximal silatrane content (both polyether-based polymers **3c, 3d** and polyester-based polymers **4c, 4d**) inhibited the fungus growth. Interestingly, this antifungal effect, which was especially noticeable at later stages, was slightly higher in the case of polyester-based polymers. Depending on the silatrane content of the polymers tested, the fungus growth inhibition rate reached 14–25% in comparison to the control. The effect of the polymeric structure of silatrane polymers on their antifungal activity is to be investigated further.

We further examined the stimulating effect of the silatrane-containing polymers on agricultural plants by studying the germination of wheat, oat and rye in the presence of silatrane-containing polymers. Evidently, the use of both polyether-based and polyester-based silatrane polymers had a positive effect on the germination percentage (GP) for all three cultures studied (Table 5).

In particular, the germination percentage of the three cultures in the silatrane polymers solutions was significantly higher than in distilled water. The mean germination time (MGT) of wheat and rye seeds in silatrane solutions was almost the same as in distilled water, while the MGT of oat seeds in silatrane solutions was slightly smaller than in distilled water. This may be due to the possible selectivity of the action of silatrane on various cultures.

## 3. Materials and Methods

### 3.1. Initial Materials

3-aminopropyltriethoxysilane (98%, ABCR, Karlsruhe, Germany), potassium tert-butanolate (97%, ABCR), triethanolamine (TEA, Himmed, Moscow, Russia), succinic anhydride (98%, ACROS, Waltham, Massachusetts, USA), (Aminomethyl)trimethylsilane (95%, ABCR), 1,1′-carbonyldiimidazole (CDI, 95%, ABCR), 1,6-Diphenylhexatriene (DPH, 98%, Sigma-Aldrich, St. Louis, MI, USA) were used as-received; triethylamine, THF, dichloromethane, benzene, DMSO, acetone, diethyl ether (Himmed, Russia) were purified by standard methods [31]; Polyglycerol (Mn = 4950 Da, Mw/Mn = 1.5) was synthesized by anionic polymerization of glycidol at INEOS RAS according to the well-known procedure [28]. Branched polyester Boltorn H40 (Mn = 7300 Da, Mw/Mn =1.25) was obtained from Perstorp Polyols AB, Perstorp, Sweden, and purified by acetone/diethyl ether precipitation method. Seeds of soft wheat (*Triticum aestivum* L., var. “Harkovskaya 46”), common oat (*Avena sativa* L., var. “Heather”) and winter rye (*Secale cereale* L., var. “Orlovskaya 9”) (GAVRISH Co., Moscow, Russia) were used without additional processing.

### 3.2. Polymer Syntheses

#### 3.2.1. Synthesis of 1-(3-aminopropyl)silatrane

The synthesis of 1-(3-aminopropyl)silatrane was performed using a modified protocol described in [32]: To a mixture of triethanolamine (15.0 mL, 16.8 g, 0.11 mol) with benzene (30 mL) was added a solution (27.3 g, 0.11 mol) of 1-(3-aminopropyl)triethoxysilane in benzene. (20 mL) and a catalytic amount (2 mg) of potassium tert-butanolate, the mixture obtained was heated to 80 °C and synthesis of silatrane was carried out for 10 h by azeotropic distillation of a mixture of benzene and ethanol while adding an equivalent amount of dry benzene to the reaction mixture. The reaction was controlled by the disappearance of hydroxy methylene proton signals in the NMR spectra of the reaction mixture. After the synthesis was completed, silatrane was not isolated but stored as a benzene stock solution of 0.544 g/mL.

^1^H NMR (500 MHz, CDCl_3_), δ: 0.24 (-Si-CH_2_-), 1.38 (-CH_2_-), 2.47 (H_2_N-CH_2_-), 2.68 (> N-CH_2_-), 3.62 (-O-CH_2_-).

#### 3.2.2. Synthesis of Polyglycerol and Boltorn H40 with a Carboxyl-Terminal Group (H40-COOH)

**Scheme 1:**
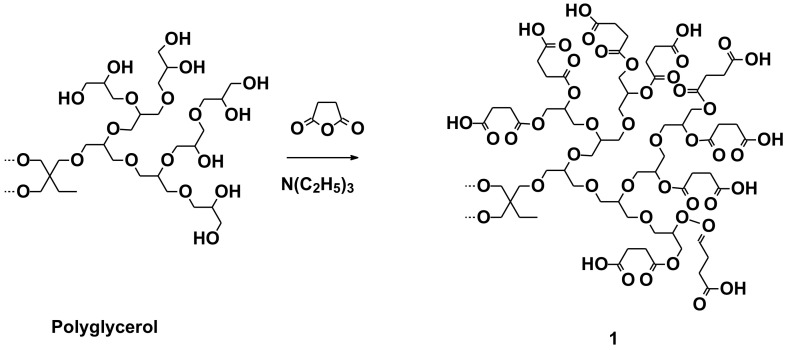
Synthesis of Polyglycerol with a carboxyl-terminal group

**Scheme 2:**
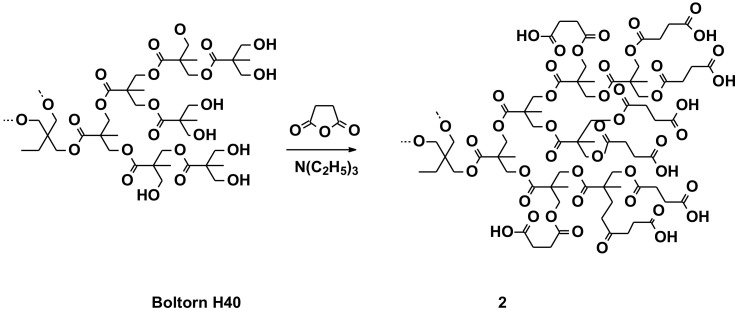
Synthesis of Boltorn H40 with a carboxyl-terminal group

Carboxyl-terminated polymers (1,3) were prepared by reaction of branched polymers with succinic anhydride, as shown on Scheme 1; Scheme 2:

In a typical procedure, 4.00 g (40 mmol) of succinic anhydride were dissolved in 75 mL of anhydrous THF in a 150 mL flask at 40 °C under vigorous stirring. Succinic anhydride being completely dissolved, the mixture of a branched polymer (2.10 g Polyglycerol or 2.85 g Boltorn H40, 25 mmol hydroxyl groups) and 4.04 g (40 mmol) of triethylamine, dissolved in 50 mL of anhydrous DMSO, was slowly added into the flask and the final mixture was reacted for 4 h. The crude product formed was precipitated twice with cold diethyl ether and dialyzed against THF (Roth “ZelluTrans” membrane (Carl Roth GmbH + Co. KG, Karlsruhe, Germany), MWCO = 1000 Da) for 48 h.

Polymer 1 ^1^H NMR (500 MHz, CDCl_3_), δ: 0.85 (CH_3_-), 1.35 (-CH_2_-), 2.51–2.80 (-CH_2_-COO-), 3.10–4.0 (-O-CH_2_-, > CH-);

Polymer 2 ^1^H NMR (500 MHz, CDCl_3_), δ: 0.85 (CH_3_-), 0.95–1.25 (-CH_3_, -CH_2_-), 2.50–2.86 (-CH_2_-COO-), 3.95–4.25 (-CH_2_-O-).

#### 3.2.3. Synthesis of Amphiphilic Polyglycerol and Boltorn H40

**Scheme 3:**
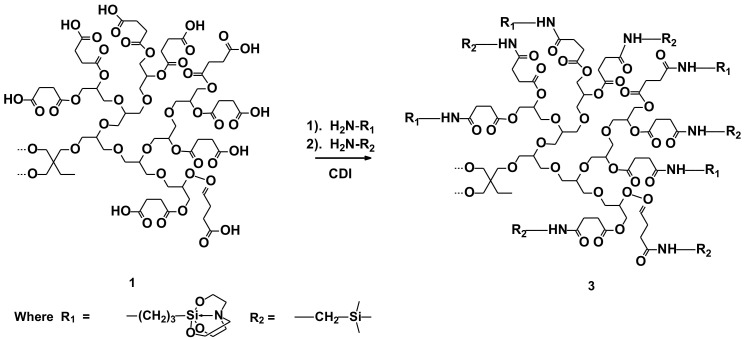
Synthesis of Amphiphilic Polyglycerol.

**Scheme 4:**
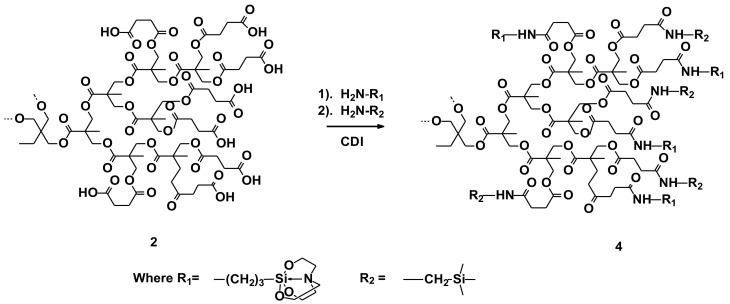
Synthesis of Amphiphilic Boltorn H40.

Organosilicon-terminated polymers (3,4) were prepared by sequential reaction of carboxyl-terminated polymers (1,2) with 1-(3-aminopropyl)silatrane and (aminomethyl)trimethylsilane, as shown on Scheme 3; Scheme 4.

In a typical procedure, 6.82 mL of stock silatrane solution was evaporated under reduced pressure, forming 3.71 g (15mmol) of dry 1-(3-aminopropyl)silatrane, which was instantly dissolved in 10 mL of THF. The carboxyl-containing polymer (5.00 g of Polymer 1 or 5.73 g of polymer 2 (25 mmol carboxyl groups)) was dissolved in 10 mL of THF, followed by adding a solution of 4.05 g (25 mmol) CDI in 10 mL of THF to it. The reaction was carried out at 40 °C for 30 min, after which solution of 1-(3-aminopropyl)silatrane in THF was slowly added to the reaction mix and reacted for 2 h; Finally, a solution of 1.03 g (10 mmol) of (aminomethyl)trimethylsilane in 10 mL of THF was slowly added to the reaction media and reacted for 2 more hours, after which THF was distilled and the polymer synthesized was purified by dialysis against THF (Roth “ZelluTrans” membrane, MWCO = 1000 Da) for 48 h.

The content of silatrane in the synthesized hybrid macromolecules was varied by changing the ratio of 1-(3-aminopropyl)silatrane to (aminomethyl)trimethylsilane.

Polymer 3 ^1^H NMR (500 MHz, CDCl_3_), δ: 0.13 (CH_3_-Si), 0.85 (CH_3_-), 0.90–1.04 (-CH_2_--Si), 1.35 (-CH_2_-), 1.92–2.14 (-CH_2_-), 2.57–2.80 (-CH_2_-COO-,-CH_2_-NH-), 3.00–3.17 (-CH_2_-NH-), 3.17–3.90(-O-CH_2_-, > CH-,-CH_2_-N <), 4.05–4.35 (-CH_2_-O-Si);

Polymer 4 ^1^H NMR (500 MHz, CDCl_3_), δ: 0.13 (CH_3_-Si), 0.85–1.25 (-CH_3_, -CH_2_-,-CH_2_-Si), 1.92–2.14 (-CH_2_-), 2.60–2.80 (-CH_2_-COO-,-CH_2_-NH-), 3.00–3.17 (-CH_2_-NH-), 3.17–3.47(-CH_2_-N <), 3.9–4.28 (-CH_2_-COO-, -CH_2_-O-Si).

### 3.3. Characterization Studies

The ^1^H NMR and ^13^C NMR spectra were recorded on a Brucker spectrometer (Bruker Corporation, Billerica, MA, USA)with operating frequency ^1^H-600.22 and 75.4 MHz, respectively MHz at 25 °C using DMSO-d6 as a solvent and TMS as an internal standard.

Gel permeation chromatography of the copolymers was carried out on a “Waters 150” chromatography (eluent–THF (1 mL/min), column–PL-GEL 5u MIXC (7.5 × 300 mm)) (Perkin Elmer, Waltham, MA, USA).

Values of the hydrophilic–lipophilic balance of the copolymers (HLB) were estimated according to the Griffin method [33]. The analytical expression of HLB of surfactant molecules is as follows:

HLB = 20 (Mh/M)(2)

where Mh and M are the molecular weights of the hydrophobic moiety and the entire molecule, respectively, for all the polymers studied, the branched macromolecular skeleton was considered hydrophobic.

The determination of the intrinsic viscosity was carried out for THF solutions at 25 °C using an Ubbelohde viscometer (DV-expert, Moscow, Russia).

### 3.4. Critical Micelle Concentration Measurements

The water used to prepare the polymer solutions in this and all other measurements was distilled and passed through a Milli-Q water purification system (Merck KGaA, Darmstadt, Germany) (surface tension of 72.5 mN/m at 20 °C).

Determination of the critical micelle concentration (CMC) was carried out by the fluorescence quenching method according to the procedure [34], using DPH as the fluorescent label. 430 nm emission fluorescence spectra were recorded using 366 nm excitation wavelength.

### 3.5. Direct and Reverse Emulsions Measurements

Preparation of direct emulsions: 4 mL of 5% solution of copolymers in methylene chloride were dispersed on ultrasound disperser UZDN-A (RKPO Ltd., Moscow, Russia) in 40 mL of water for 30 s, ultrasound power 15 W.

To obtain reverse emulsions, 0.1 mL of water were sonicated in 10 mL of 5% solution of copolymers in methylene chloride for 30 s, an ultrasound power of 15 W. The average size of the emulsion droplets was determined by the Photocor complex photon correlation spectrophotometer (Photocor Ltd., Moscow, Russia).

### 3.6. Contact Angle Measurements

The drops of the polymer solutions were formed over borosilicate microscope cover glass slides (Borosilicate 3.3, Kemtech America Inc., USA) or Parafilm M (PM-999, Bemis Company Inc., St. Louis, MI, USA) placed upon microscope glass slides.

The contact angle between the polymer solutions and solid surfaces was measured by the sessile drop method on a “Tracker” automatic drop shape analyzer (Teclis-Scientific, Civrieux d’Azergues, France) at 25 °C and about 100% relative humidity in drops 100 s after their formation. The concentration of water solutions for each polymer was equal to 2 CMC.

### 3.7. Antifungal Activity Studies

The study of the antifungal activity of silatrane-containing polymers and low molecular weight 1-(3-aminopropyl)silatrane was performed using the standard procedure of radial growth of colonies [35] of phytopathogenic fungus *Verticillium dahliae*. The degree of inhibition of the fungal colony growth was determined as the ratio of the diameter of the colony grown in the presence of the silatrane-containing compounds to the diameter of the colony grown on a nutrient medium without these compounds. In all the biological experiments, 4.5 × 10^−7^ mol L^−1^ of silatrane was used since this concentration had been shown [21,22] to be optimal for elucidating the physiological activity of silatranes.

### 3.8. Seed Germination Studies

Seed germination experiments were carried out for 10 days according to the rules of the International Seed Testing Association [36]. Germination percentage (GP) and mean germination time (MGT) was calculated as follows:

GP = total number of seeds germinated/total number of seeds × 100(3)

MGT = Σ (nt)/Σ n(4)

where n = the number of seeds newly germinated at time t; t = days from the beginning of the germination test.

## 4. Conclusions

The results obtained in this study show the possibility of creating biodegradable and biocompatible amphiphilic polymers containing silatrane groups. By varying the structure and content of silatrane fragments in such polymers, one can change their surface properties. The effect of the ratio of hydrophilic and hydrophobic organosilicon structures on the surface activity and biological activity of macromolecules, as well as the effect of the chemical structure of macromolecules on these properties, is shown for the first time. In particular, the critical micelle concentrations were determined, the effect of the structure of polymers on the wetting of glass and parafilm with aqueous solutions of polymers was described, and the aggregation stability of emulsions was studied. Finally, the effect of the structure of polymers on their antifungal activity and stimulation of seed germination was investigated. It was shown that silatrane-containing polymers noticeably slow down the development of the phytopathogenic fungus *Verticillium dahliae* and affect the germination of wheat, oat, and rye seeds. We believe that the biological effects of polymer silatranes are related to their polymer backbone structure, but further studies are needed to study these effects in detail.

## Figures and Tables

**Table 1:** Composition and molecular weights of silatrane-containing polymers.

Polymer	Yield,%	Ratio of R_1_ to R_2_ Substituents ^1^		Mn ^2^	Mw/Mn ^2^	Intrinsic Viscosity dL/g (25 °C)
		Calculated	Measured			
3a	96	10:90	10:90	8150	2.3	4.445
3b	94	30:70	28:72	7950	2.6	4.622
3c	96	60:40	58:42	8300	2.8	4.818
3d	93	90:10	90:10	8450	3.1	5.136
4a	97	10:90	9:91	8250	2.7	1.554
4b	98	30:70	29:71	7600	3.2	1.713
4c	96	60:40	56:44	8700	3.1	1.932
4d	93	90:10	88:12	8900	3.4	2.282

^1^ ratios of R_1_ to R_2_ substituents in branched amphiphilic polymers (Scheme 1; Scheme 2), determined from monomer load (calculated) and from NMR spectra of the polymers (measured);.^2^ obtained by GPC.

**Table 2:** The hydrophilic/lipophilic balance (HLB) and CMC values of silatrane-containing polymers.

Polymer	HLB	CMC, M	Polymer	HLB	CMC, M
3a	1.6	1.4 × 10^−4^	4a	1.3	9.8 × 10^−5^
3b	4.2	7.1 × 10^−3^	4b	4.0	4.6 × 10^−4^
3c	7.6	5.6 × 10^−2^	4c	6.9	8.1 × 10^−3^
3d	10.5	7.1 × 10^−1^	4d	9.7	1.4 × 10^−2^

**Table 3:** The contact angle values of silatrane-containing polymer solutions on different substrates. (θ, grad).

Polymer	Glass	Parafilm	Polymer	Glass	Parafilm
3a	30	43	4a	32	37
3b	26	56	4b	27	51
3c	23	71	4c	19	63
3d	18	86	4d	22	71

**Table 4:** Suppression of the radial growth of *Verticillium dahliae* colonies by silatrane-containing polymers **3c**–**4d**.

Polymer	Radial Size of *Verticillium dahlia* Colonies (cm), after Time t, Days					
	2	3	4	5	6	7
Water (control)	1.7	2.4	3.2	4.1	4.8	5.7
3c	1.3	2.0	2.8	3.3	4.0	4.9
3d	0.5	0.8	1.2	1.6	2.2	2.8
4c	1.2	1.7	2.3	3.0	3.5	4.3
4d	0.6	0.9	1.3	1.6	2.0	2.3

**Table 5:** Germination parameters of some crops in solutions of silatrane polymers.

Polymer	Wheat		Oat		Rye	
	GP	MGT	GP	MGT	GP	MGT
− ^1^	78.3 ± 0.6	3.9± 0.03	77.67 ± 0.57	3.81 ± 0.04	69.00 ± 1.00	4.43 ± 0.06
3d	91.0 ± 1.0	3.9± 0.07	95.33 ± 0.57	3.50 ± 0.15	88.33 ± 0.57	4.43 ± 0.07
4d	89.0 ± 1.0	3.9 ± 0.07	97.00 ± 1.00	3.59 ± 0.16	92.33 ± 0.57	4.36 ± 0.02

^1^ distilled water (control).

## Data Availability

The data presented in this study are available on request from the corresponding author.

## Sample Availability

Samples of the compounds are not available from the authors.
